# Comparative proteogenomics profiling of non-small and small lung carcinoma cell lines using mass spectrometry

**DOI:** 10.7717/peerj.8779

**Published:** 2020-04-23

**Authors:** Jingyu Wu, Zhifang Hao, Chen Ma, Pengfei Li, Liuyi Dang, Shisheng Sun

**Affiliations:** College of Life Science, Northwest University, Xi’an, China

**Keywords:** Non-small cell lung cancer, Small cell lung cancer, Transcriptomics, Proteomics, Bioinformatics, Proteogenomics, Mass spectrometry

## Abstract

**Background:**

Evidences indicated that non-small-cell lung cancer (NSCLC) and small-cell lung cancer (SCLC) might originate from the same cell type, which however ended up to be two different subtypes of lung carcinoma, requiring different therapeutic regimens. We aimed to identify the differences between these two subtypes of lung cancer by using integrated proteome and genome approaches.

**Methods and Materials:**

Two representative cell lines for each lung cancer subtype were comparatively analysed by quantitative proteomics, and their corresponding transcriptomics data were obtained from the Gene Expression Omnibus database. The integrated analyses of proteogenomic data were performed to determine key differentially expressed proteins that were positively correlated between proteomic and transcriptomic data.

**Result:**

The proteomics analysis revealed 147 differentially expressed proteins between SCLC and NSCLC from a total of 3,970 identified proteins. Combined with available transcriptomics data, we further confirmed 14 differentially expressed proteins including six known and eight new lung cancer related proteins that were positively correlated with their transcriptomics data. These proteins are mainly involved in cell migration, proliferation, and invasion.

**Conclusion:**

The proteogenomic data on both NSCLC and SCLC cell lines presented in this manuscript is complementary to existing genomic and proteomic data related to lung cancers and will be crucial for a systems biology-level understanding of the molecular mechanism of lung cancers. The raw mass spectrometry data have been deposited to the ProteomeXchange Consortium via the PRIDE partner repository with the dataset identifier PXD015270.

## Introduction

Lung cancer is the leading cause of cancer death in the world. According to the cancer statistics in 2018, the number of new cases and deaths reached the top (2,093,876 and 1,761,007) in the world. The incidence and mortality of lung cancer presented for 11.6% and 18.4% in the world (Bray et al., 2018). World Health Organization (WHO) classifies lung cancer into two broad histological subtypes: non-small cell lung cancer (NSCLC) which is the cause of about 85% of cases, and small cell lung cancer (SCLC), which accounts for the remaining 15%. Compared to NCLC, cells of SCLC are smaller but more likely to spread to other tissues or organs. Due to the aggressiveness of SCLC and its poor prognosis, patients with SCLC usually have much shorter life expectancy compared to most cases of metastatic NSCLC (Blandin Knight et al., 2017).

Considering the huge differences between NSCLC and SCLC in their diagnosis and therapeutic regimens, it is important for us to understand the essential differences between these two subtypes of lung cancers. Comparative analysis of NSCLC and SCLC have been performed using different high-throughput approaches, such as genomics, transcriptomics and proteomics. A genome-wide allelotyping study in 2001 showed that tumor suppressor genes were significantly different between NSCLC and SCLC. In an integrated COSMIC database created by 2016 (Forbes et al., 2015), TP53, RB1, EGFR and KRAS genes were found to be the most prone to mutations in the two lung cancer subtypes (Zhang et al., 2017). Recently, proteogenomic analysis has become a powerful tool for cancer research and several studies on lung cancer have been reported. In a recent study, Sharpnack et al. (2018) established a novel analysis about the co-relation between mRNA and proteins and predicted 51 potential biomarkers for lung cancer. Treue et al. (2019) reported EGFR-mutated NSCLC with whole exome sequencing data, phosphorylated protein data and computational models, and identified three potential biomarkers for therapy targets. APOBEC, a DNA deaminase, was identified as a gene of mutational heterogeneity which might be associated with tumour migration (Roper et al., 2019). Although a large amount of work have been done on NSCLC and SCLC, the essential differences between NSCLC and SCLC remain to be fully characterized (Sutherland et al., 2011).

Here, we comparatively analysed the protein expressions of the NSCLC cell lines (A549 and H1975) and the SCLC cell lines (H446 and H69) using quantitative proteomics to identify differentially expressed proteins (DEPs) between these two subtypes of lung cancers. In addition, their transcriptomic data were also downloaded from Gene Expression Omnibus (GEO) public database. Various bioinformatic approaches, such as Gene Ontology enrichment, Kyoto Encyclopedia of Genes and Genomes (KEGG) pathway analysis, and protein–protein interaction (PPI) network integration were applied for the investigation of the DEPs (and genes). With the comparison of proteomics and genomics data, we pinpointed several gene/protein candidates with potential biological significances, of which the possible functions in lung cancer were further discussed. To our knowledge, this is the first study to investigate differences between NSCLC and SCLC by integrated proteomics and transcriptomics analyses. Our data may provide potential biological candidates for further study of lung cancer as well as to understand the molecular differences between NSCLC and SCLC.

## Methods and Materials

### Cell culture

Two NSCLC cell lines (H1975, A549) and two SCLC cell lines (H69, H446) were obtained from American Type Culture Collection (ATCC). The cell lines were cultivated in the RPMI-1640 culture medium supplemented with 10% fetal bovine serum (FBS) and 1% Penicillin-Streptomycin Solution in a humidified incubator at 37 °C and 5% CO_2_. The NSCLC cell lines were cultivated with adherent method and SCLC cell lines were cultivated in suspension method. After removing the medium, the cells were washed with phosphate buffered saline (PBS, pH 7.4) buffer and then lysed directly with 8 M urea/1 M NH_4_HCO_3_ solution (Sun et al., 2014). Lysates were briefly sonicated until the solutions became clear. Protein concentrations were determined by BCA protein assay reagent (Beyotime Biotechnology, Shanghai).

### Protein extraction and trypsin digestion

The protein extraction from cell lines and protein digestion were performed as described previously with minor modifications (Sun et al., 2016). Briefly, lung cancer proteins were denatured in the 8M urea /1M NH_4_HCO_3_ buffer, sonicated by Ultrasonic Cell Distribution System, reduced by 5 mM DTT at 37 °C for 1 h and alkylated by 15 mM IAM at room temperature in the dark for 30 min. The solutions were diluted two-fold with deionized water, and the proteins were digested with sequencing grade trypsin (Promega, Madison WI; protein; enzyme, 100:1, w/w) at 37 °C for 2 h with shaking. The solutions were further diluted by four-fold, and additional trypsin (protein; enzyme, 100:1, w/w) were added and incubated at 37 °C overnight with shaking. Then samples were centrifuged at 15,000 g for 10 min to remove cell residues and desalted with HLB column (Waters, Milford, MA). The peptides were eluted by 60% ACN/0.1% TFA.

### LC-MS/MS analysis

Each sample underwent triplicate LC-MS/MS runs on an Orbitrap Fusion Lumos mass spectrometer (Thermo Fisher Scientific, Bremen, Germany). Peptides were separated on a nano Easy-LC system with a 75 µm ×  15 cm Acclaim PepMap100 separating column protected by a two cm guard column (Thermo Scientific, Fair Lawn, NJ). The mobile phase flow rate was 300 nL/min and consisted of 0.1% formic acid in water (A) and 0.1% formic acid 80% acetonitrile (B). The gradient profile was set as follows: 3–7% B for 2 min, 7–35% B for 83 min, 35–68% B for 20 min, 68–100% B for 5 min and equilibrated in 100% B for 10 min. MS analysis was performed using a mass spectrometer. The spray voltage was set at 2.3 kV. Orbitrap MS1 spectra (AGC 4 ×  10^5^) were collected from 350–1,800 m/z at a resolution of 60 K followed by data dependent HCD MS/MS (resolution 15,000, collision energy 30%) using an isolation width of 1.6 Da. Charge state screening was enabled to reject unassigned and singly charged ions. A dynamic exclusion time of 45 s was used to discriminate against previously selected ions.

### Database search and label-free quantitation

Mass spectrometric data were searched against the UniProt/SwissProt human proteome database (20,341 proteins, downloaded from http://www.uniprot.org on May 25th, 2018) using MaxQuant (version 1.6.3.3) (Cox & Mann, 2008). The precursor and fragment ion mass tolerance were set to 5 ppm and 20 ppm, respectively. The enzyme specificity was set to trypsin, and two missed cleavages were allowed. The minimum peptide length was set to 7 amino acids. Cysteine carbamidomethylation was set as fixed, and methionine oxidation and N-terminal acetylation were set as variable modifications. A maximum of 5 modifications per peptide was allowed. The false discovery rates (FDR) of both peptide and protein identification were set to 1% (Tyanova, Temu & Cox, 2016). The “Match between runs” based on the accurate m/z and mass spectra retention time was used with a min 0.7 match time window and min 20 alignment time window (Bielow, Mastrobuoni & Kempa, 2016). For the calculation of the protein abundances, label-free quantitation (LFQ) was performed with an LFQ minimum ratio count of two. The normalization of label-free quantitation (LFQ) was performed based on the total intensities of all detected peaks in each LC-MS data, which is a default setting in the MaxQuant and has been described in detail in Cox’s research (Cox et al., 2014). The medium of normalized ratios from non-modified peptides were used for the protein quantitation.

### Transcriptomic microarray data and difference analysis

An authoritative public cancer database of 947 human cancer cell lines from CCLE (Cancer Cell Line Encyclopedia) (Barretina et al., 2012) were downloaded from Microarray dataset GSE36133 which was obtained from GEO database (http://www.ncbi.nlm.nih.gov/geo/) (Edgar, Domrachev & Lash, 2002), and based on the Affymetrix Human Genome U133 Plus 2.0 Array platform (Mitra et al., 2012). For data pre-processing, the probe-level data in CEL files were converted into expression measures by using the affy package in R language (Gautier et al., 2004), and then was subjected to background correction and quartile data normalization by using robust multiarray average (RMA) algorithm. Each probe was mapped to its corresponding gene using Bioconductor annotation function of R language (Gentleman et al., 2004). The probes corresponding to no gene or more than one gene were deleted. When there were several probes for one gene, the highest *P*-value of these probes was used as the expression value of the gene.

### Determination and hierarchical clustering analysis of DEGs

Linear Models for Microarray Analysis package in R language was employed to screen DEGs between NSCLC samples and SCLC samples. The strict thresholds were set at fold-change (—log_2_ FC—) ≥1 and *P*-value <0.01. The screened DEGs underwent two-way hierarchical clustering analysis by using the pheatmap package in R language.

### Gene ontology and pathway analysis

Gene Ontology (GO) analysis was undertaken for the significantly different expressed genes and proteins in order to find the unique biological process, cellular component and molecular function. GO enrichment analysis was performed by DAVID (https://david.ncifcrf.gov/) followed by the ggplot2 R language package (Huang da, Sherman & Lempicki, 2009). Kyoto Encyclopedia of Genes and Genomes (KEGG) pathway was performed by using ClueGO plug-in and Cluepedia of Cytoscape software (Otasek et al., 2019) to display the multiple biological pathways according to different express genes and proteins (Bindea et al., 2009; Shannon et al., 2003). KEGG pathway enrichment analysis was performed to search for the associated important pathway information and key proteins and genes. In this study, two-sided hypergeometric test and Benjamini–Hochberg were used to calculate *p*-value. A pathway with adjusted *P*-value <0.05 was regarded as the significant pathway.

### PPI establishment and key proteins analysis

Protein interaction was constructed using the significantly different expressed genes and proteins. The STRING website (https://string-db.org/) was used to query whether the proteins interacted with other proteins with combined score output (Snel et al., 2000). In this study, the interaction score ≥0.4 were used and performed by Cytoscape.

### Selection of core proteins by MCODE and Reactome enrichment

Among these significant DEPs, the highly interconnected proteins in PPI network were selected by the MCODE plug-in of Cytoscape. MCODE (Bandettini et al., 2012) was the most co mMon module in Cytoscape which filtered with k-score. In this study, we used Haircut, node score cut-off (0.2), K-core (2), and Max.Depth (100) for clustering core proteins. The functional analysis of each module was enriched by Reactome (https://www.reactome.org/) with the significance threshold of *P*-value<0.05, FDR<0.01 (Fabregat et al., 2016).

### Correlation analysis by Pearson Linear regression

The correlation between proteins and mRNA intensities in two subtypes of lung cancer cell lines was calculated by Pearson correlation analysis in “Corrplot” R package. The correlation coefficient which higher than 0.4 was considered as positive correlation.

## Results

### Proteomic profiling of NSCLC and SCLC cells

**Figure 1:**
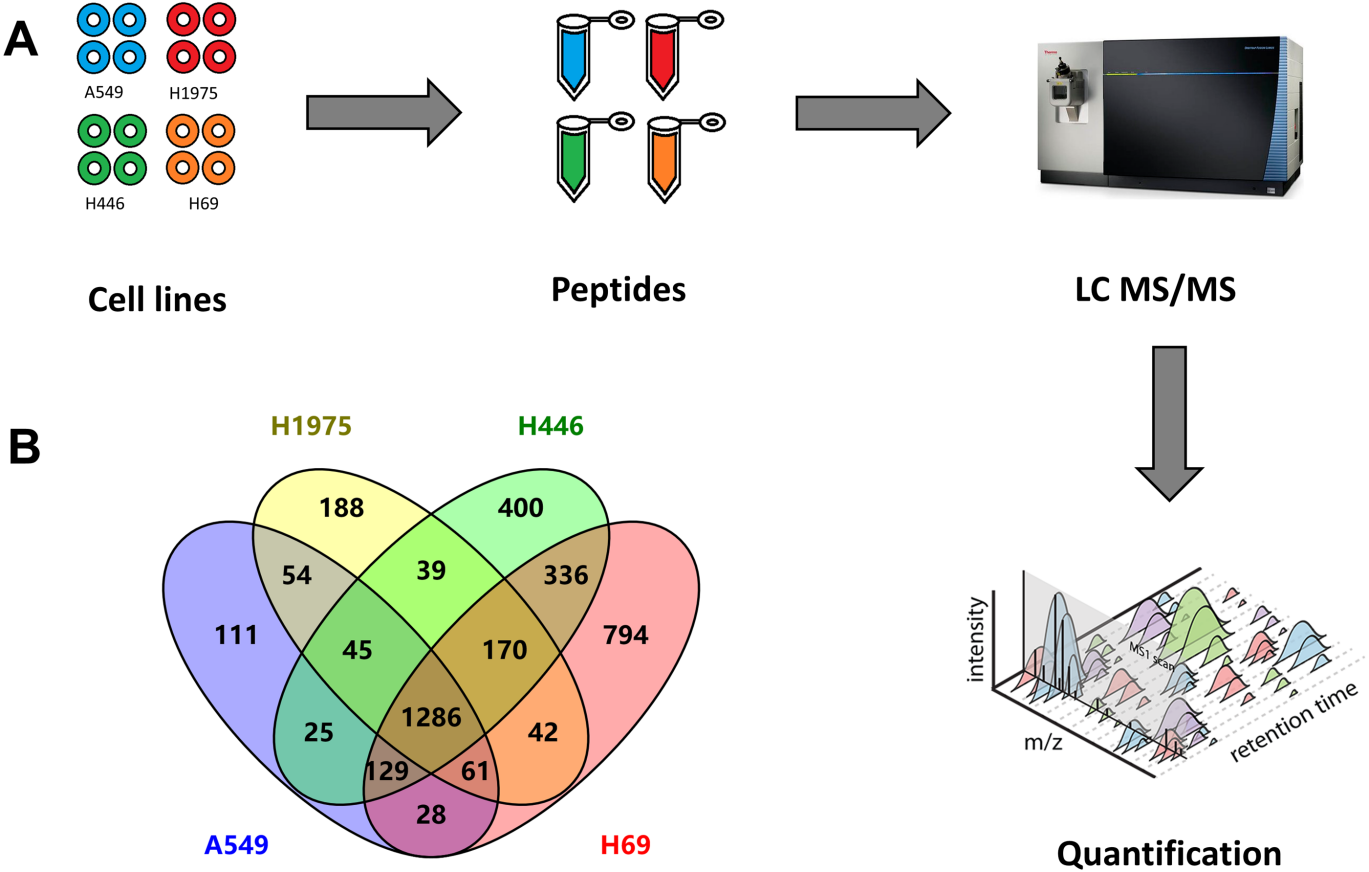
Quantitative proteomic analysis of two NSCLC cells (A549, H1975) and two SCLC cell lines (H69, H1975). (A) Workflow of this study, including the sample preparation, mass spectrometry data generation, and label-free qualification of proteins among cell lines. (B) Venn diagram of identified proteins among four cell lines.

In this study, four different cell lines were analysed by quantitative proteomics to investigate the DEPs between NSCLC and SCLC (Fig. 1A). Among these four cell lines, A549 and H1975 represent two major gene mutation types of adenocarcinoma NSCLC. According to the cBioPortal database (Cerami et al., 2012), the most mutation genes in NSCLC were TP53 (58%), KRAS (32%), EGFR (15%), and PIK3CA (10%). The A549 cell line has KRAS gene mutation and H1975 cell line has TP53, EGFR, PIK3CA gene mutations. Another two cell lines, H446 and H446 own TP53 and RB1 gene mutations and represent semi suspension and suspension SCLC cell lines, respectively.

From these four cell lines, we totally identified 3,970 proteins, including 1,739 proteins from A549 cells, 1,885 proteins from H1975 cells, as well as 2,429 and 2,845 proteins from H446 and H69 cells, respectively (Fig. 1B and Table S1). Among these proteins, 1,286 proteins were identified from all four cell lines, while 336 proteins were specifically identified in both SCLC cell lines, and 54 proteins specifically in two NSCLC cell lines.

### Identification of differentially expressed proteins by quantitative proteomics

**Figure 2:**
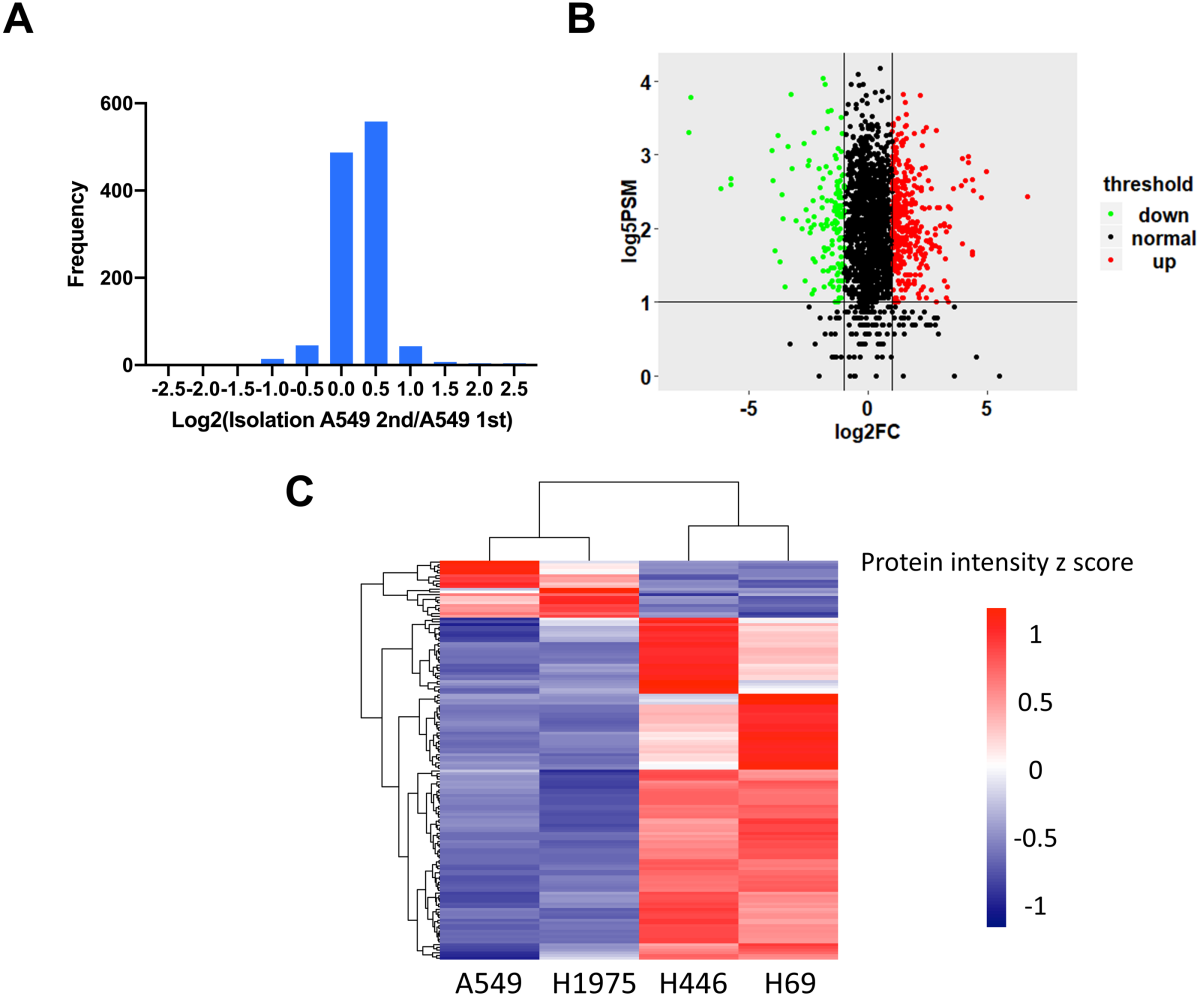
Identification of differentially expressed proteins between NSCLC and SCLC. (A) Reproducibility of four cell lines in technical duplicates. The four lung cancer cell lines were analysed twice by LC-MS/MS. (B) Volcano plot of the distribution of differentially expressed proteins. (C) Cluster analysis of differentially expressed proteins with *z*-scored protein abundance among four cell lines.

To identify the DEPs between NSCLC and SCLC cell lines, we first determined the data reproducibility in all cell lines. The quantification results between duplicate LC-MS/MS analysis of the proteins from four cell lines indicated that 97.6 ± 1.2% of quantitative proteins were within two-fold changes (Fig. 2A and Table S2). A two-fold change was then used as the filter for identifying changed proteins in the following analysis. In order to further increase the quantitation accuracy, we also filtered the selected DEPs by PSMs ≥5. Based on these criteria, a total of 147 proteins were identified to be significantly changed between NSCLC and SCLC cells, including 126 proteins up-regulated and 21 proteins down-regulated in SCLC cancer cell lines (Fig. 2B and Table S3). The gierarchical clustering heat map (Fig. 2C) showed the intensities of these 147 proteins within the four cell lines, which can be divided into three main groups based on their expression patterns.

### Gene ontology analysis of differentially expressed proteins

**Figure 3:**
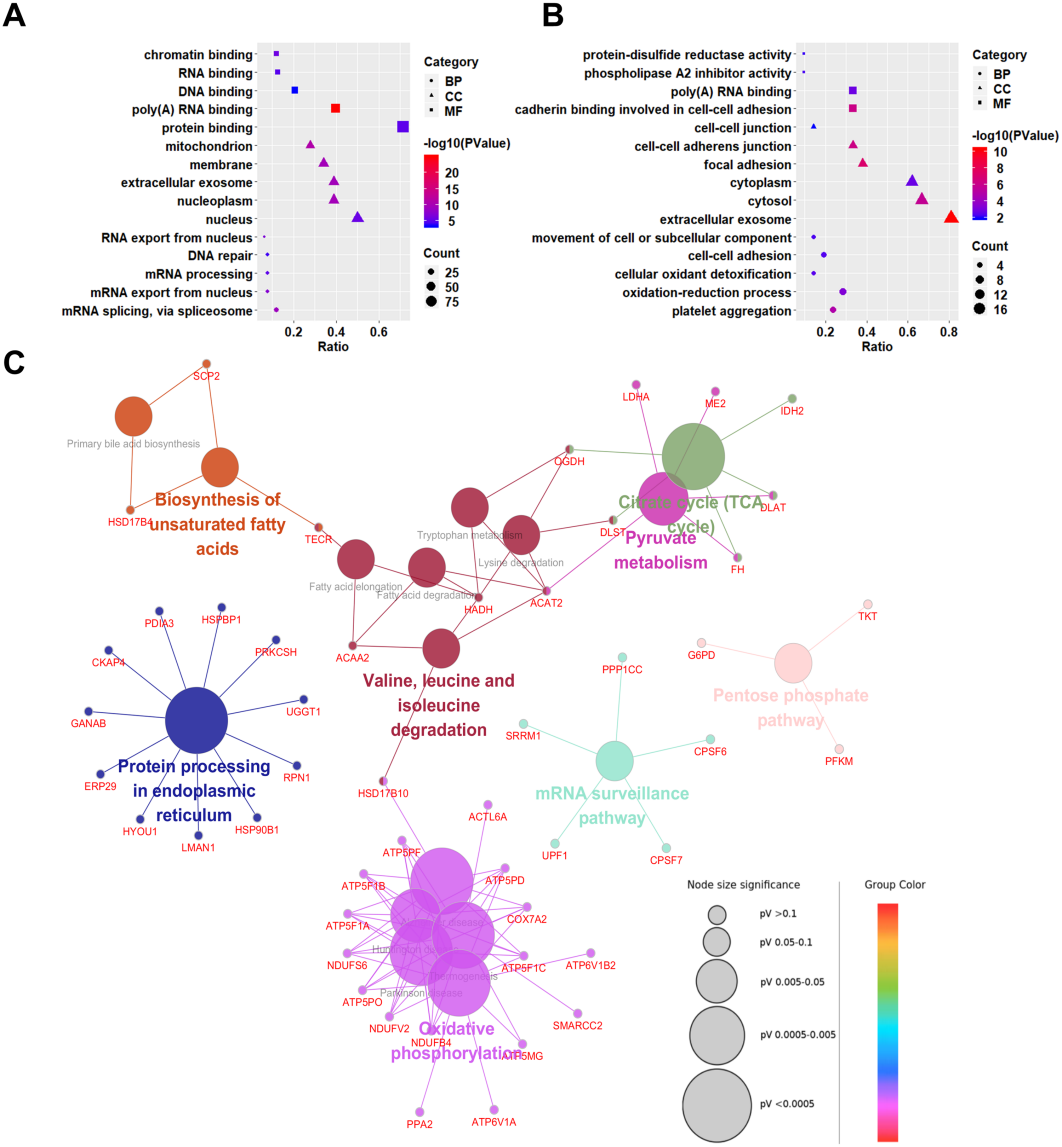
Gene Ontology (GO) and KEGG pathway analyses of differentially expressed proteins between NSCLC and SCLC cell lines. (A) GO enrichment analysis of up-regulated proteins in SCLC. (B) GO enrichment analysis of down-regulated differentially expressed proteins in SCLC. (C) KEGG Pathway analysis of all differentially expressed proteins.

To explore the biological significances of the identified DEPs, the Gene Ontology analysis was performed. We first focused on the up-regulated proteins in SCLC cell lines (Fig. 3A). Regarding to the biological processes (BP), the up-regulated proteins were mainly involved in the process of mRNA splicing, via spliceosome, mRNA export from nucleus, mRNA processing, DNA repair, and RNA export from nucleus. For the cellular component (CC) category, the up-regulated proteins were localized in the nucleus, nucleoplasm, extracellular exosome, membrane, and mitochondrion. In addition, the up-regulated proteins were significantly enriched in the molecular functions (MF) of the protein binding, poly(A) RNA binding, DNA binding, RNA binding, and chromatin binding.

Down-regulated proteins in SCLC cell lines participated in the biological processes of the platelet aggregation, oxidation–reduction process, cellular oxidant detoxification, cell–cell adhesion, and movement of cell or subcellular components (Fig. 3B). They were mainly enriched in the cellular components of the extracellular exosome, cytosol, cytoplasm, focal adhesion, cell–cell adherents’ junction, and cell–cell junction. In terms of molecular functions, the down-regulated proteins were enriched in the cadherin binding involved in cell–cell adhesion, poly(A) RNA binding, phospholipase A2 inhibitor activity, and protein-disulphide reductase activity.

### Kyoto Encyclopedia of Genes and Genomes (KEGG) pathway analysis

To predict the relevant molecular interaction, reaction and relation networks of DEPs, the KEGG pathway analysis were conducted using ClueGO plug-in from Cytoscape software. with the Kappa score ≥0.4 as a cut-off, 17 pathways were significantly enriched (*P*-value < 0.05). Most of the DEPs were enriched in the citrate cycle (TCA cycle), pyruvate metabolism, valine, leucine and isoleucine degradation, biosynthesis of unsaturated fatty acids, protein processing in endoplasmic reticulum, oxidative phosphorylation, mRNA surveillance pathway, and pentose phosphate pathway (Fig. 3C).

### PPI network construction and core protein selection

**Figure 4:**
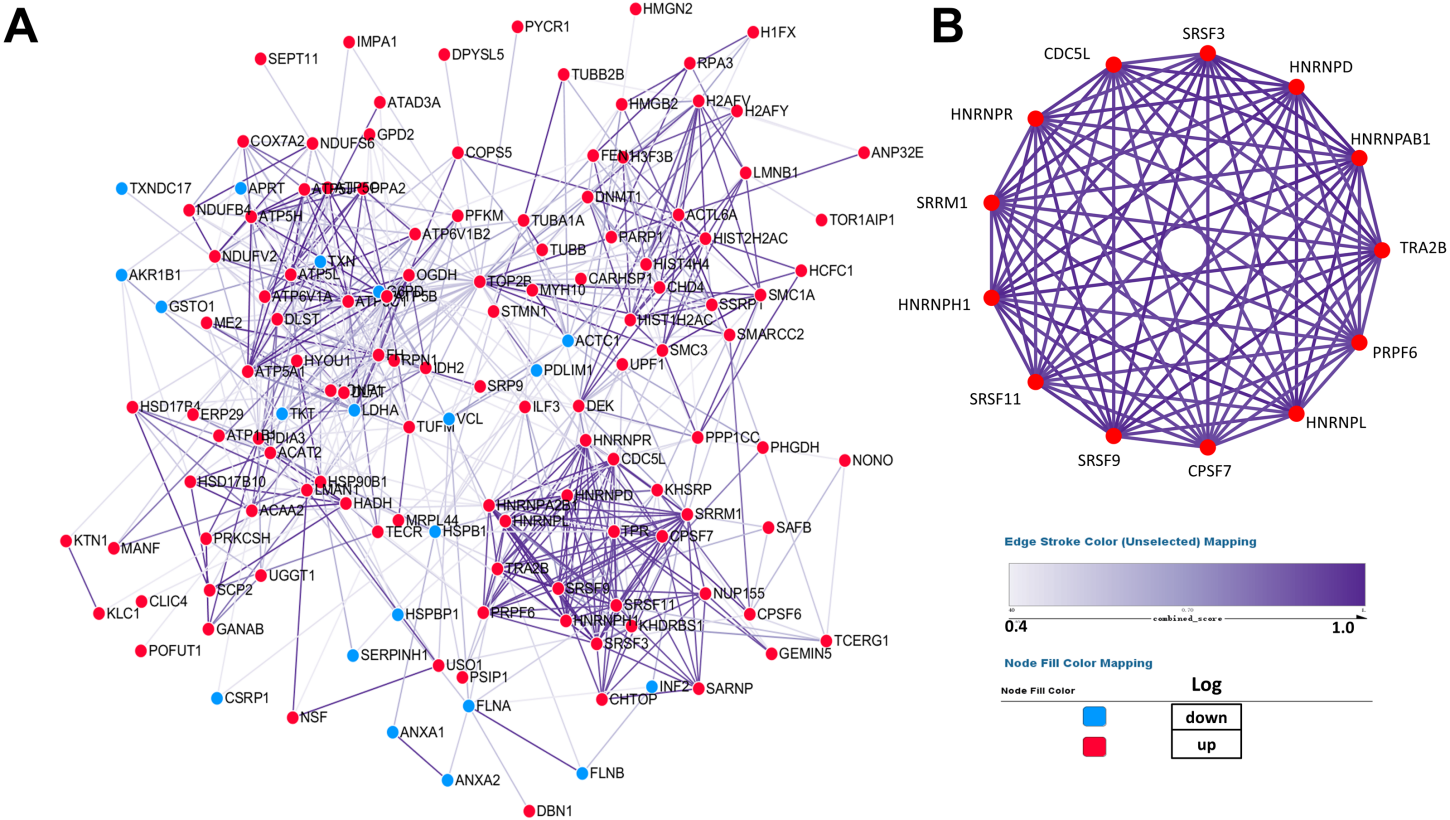
Establishment of protein–protein interaction network. According to the results from the STRING database, the tightness of the relationship among DEPs clearly increases as the colour deepens, which were derived from the real experiments and statistical analysis. (A) Network of 147 differentially expressed proteins. (B) Network of highly interconnected proteins.

PPI (protein–protein interaction) network was performed by Cytoscape to investigate the biological and physiological connections among DEPs (Fig. 4A). Compared with NSCLC, the number of up-regulated proteins in SCLC was more than that of down-regulated proteins and some DEPs showed high degree of interactions, which illustrated that the core proteins in network play a crucial role in lung cancer.

To better demonstrate the interconnections of proteins in the interaction network, proteins involved in RNA processing were displayed (Fig. 4B), which include the processing of capped intron-containing pre-mRNA(r), spliceosome(k), RNA polymerase ii transcription(R), mRNA surveillance pathway(K), and processing of capped intron less pre-mRNA(R).

### Determination of key proteins by combined use of proteomics and transcriptomics

**Figure 5:**
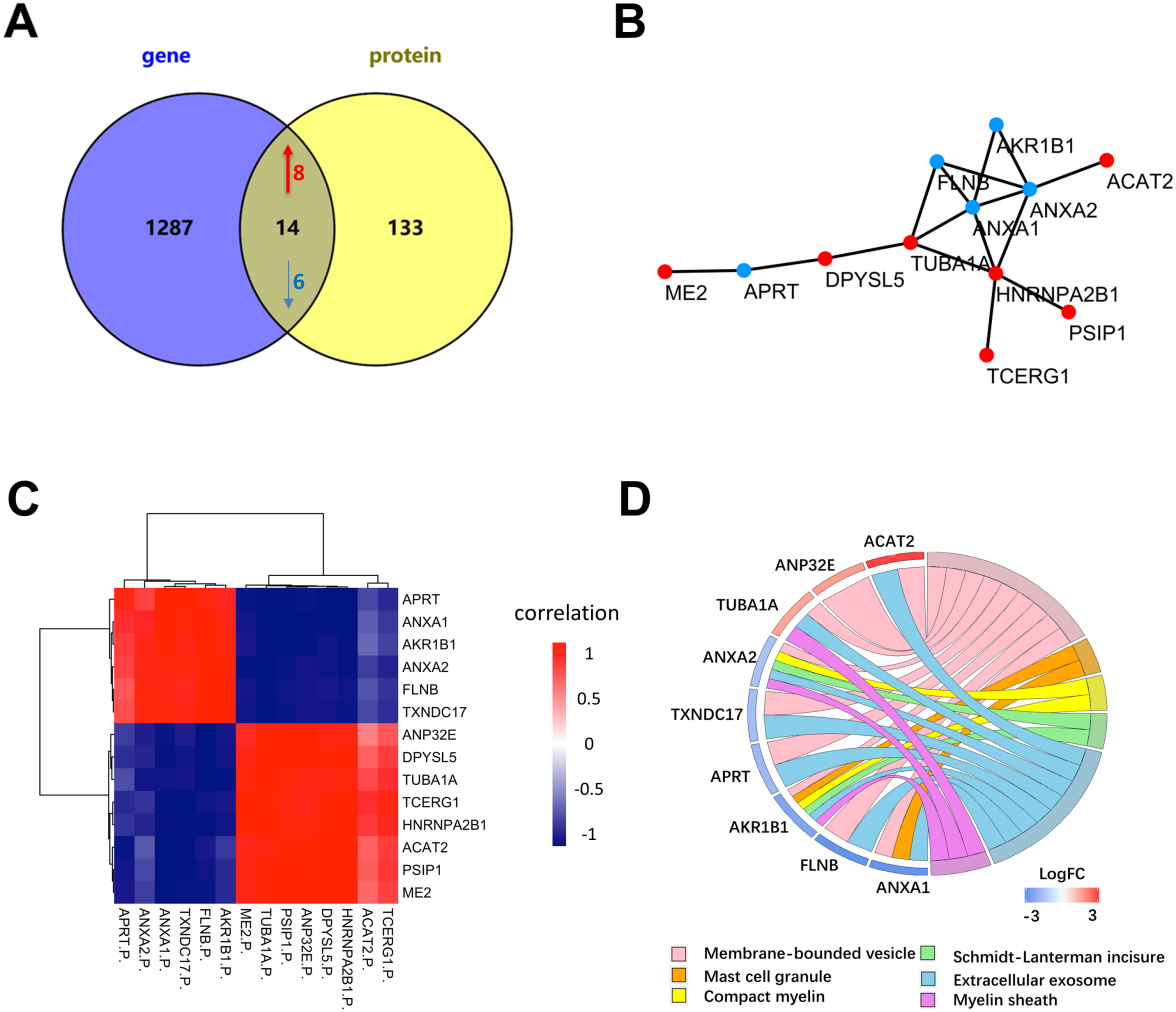
Differentially expressed proteins between NSCLC and SCLC cell lines at both the mRNA and protein level. (A) The total differentially expressed proteins and genes in proteogenomic data. (B) PPI network of 14 differentially expressed proteins and genes in proteogenomic data. (C) Co-relation of 14 differentially expressed proteins between mRNA and proteins. (D) Reactome pathway analysis of 14 differentially expressed proteins.

To further determine the key proteins between NSCLC and SCLC cells, we compared the proteomic data with published microarray-based transcriptomic data from the same cell lines (Barretina et al., 2012) (Fig. 5A). Among 1,220 quantitative proteins, 1,128 (92.5%) matched their corresponding mRNA quantitation data. The previous study (De Sousa Abreu et al., 2009) showed that the correlation between mRNA and proteins was approximately 0.4 in prokaryotes and eukaryotes in general and much lower in other specific species. By further filtering the results by the correlation coefficient higher than 0.4, there were a total of 14 proteins were considered to have a highly positive correlation with their transcriptomic data (Fig. 5B, Table 1 and Table S4). The six proteins (ANXA1, ANXA2, FLNB, ME2, HNRNPA2B1, APRT) have been reported in recent studies and were further validated by our proteogenomic approach. Through our analysis, we also found other eight proteins (ACAT2, PSIP1, TCERG1, DPYSL5, TUBA1A, AKR1B1, ANP32E, and TXNDC17) that have been associated with other cancers, but not in lung cancer. According to the diagram, ANXA1 and ANXA2 were the centre of relationship network.

**Table 1:** The profiling of differentially expressed proteins and mRNA.

**Protein name**	**Description**	**Gene name**	**Ratio**	**PSM**
P04083	Annexin A1	ANXA1	0.072	761
P07355	Annexin A2	ANXA2	0.314	1,364
O75369	Filamin-B	FLNB	0.106	1,816
Q9BW1	Acetyl-CoA acetyltransferase 2	ACAT2	7.92	112
O75475	PC4 and SFRS1-interacting protein	PSIP1	15.25	266
O14776	Transcription elongation regulator 1	TCERG1	7.166	101
P07741	Adenine phosphoribosyl transferase	APRT	0.262	160
Q9BP6	Dihydropyrimidinase-related protein 5	DPYSL5	3.058	173
Q71U36	Tubulin alpha-1A	TUBA1A	2.969	244
P23368	NAD-dependent malic enzyme 2	ME2	3.614	252
P22626	Heterogeneous nuclear ribonucleoproteins A2/B1	HNRNPA21	2.924	1,600
P15121	Aldose reductase	AKR1B1	0.179	461
Q9BTT0	Acidic leucine-rich nuclear phosphoprotein 32 family member E	ANP32E	3.249	61
Q9BR2	Thioredoxin domain-containing protein 17	TXNDC17	0.294	109

Reactome enrichment indicated that nine of these 14 proteins were enriched in membrane-bounded vesicle, mast cell granule, compact myelin, Schmidt-Lanterman incisures, extracellular exosome and myelin sheath (Fig. 5D). The biological significances of these proteins were further discussed in the following section.

## Discussion

Lung cancer is the leading cause of cancer death in the world. Due to the different pathway regulations, NSCLC and SCLC required different therapy regimens. The aim of this study was to investigate the DEPs between NSCLC and SCLC, which could be helpful in understanding the development of the disease and the search of possible treatment targets. The quantitative proteomics approach was used to identify the proteins differentially expressed between NSCLC and SCLC by analysing two representing cell lines per subtype. The GEO data were then used as a supplement for the data analysis and determination of pivotal proteins. Among 3,970 proteins identified from four cell lines, a total of 147 were determined to be differentially expressed between two lung cancer subtypes. The majority of proteins showed no difference between NSCLC and SCLC, implying the same or similar origin of these two lung cancer types (Oser et al., 2015).

The results of GO and KEGG pathway analyses showed that these DEPs were enriched in several different pathways, biological processes, cellular components and molecular functions. Based on the GO analysis, the nucleus, DNA binding, as well as DNA repair may be involved in cell replication, and protein binding, membrane might be involved in cell recognition. Therefore, these up-regulated proteins in SCLC might be associated with cell proliferation and recognition. Additionally, the down-regulated proteins might be associated with cell adhesion and migration such as cell–cell junction, movement of cell or subcellular component.

According to the results from the PPI network, we found some highly interconnected proteins. Among these proteins, the results of enrichment revealed that mRNA processing was closely associated with cancer initiation and development, which were consistent with previous studies (Yoshimi & Abdel-Wahab, 2017). Numerous studies have validated that splicing factors have a prominent contribution to cancer, which can impact splicing of oncogenes and tumour suppressors. (Grosso, Martins & Carmo-Fonseca, 2008; Venables, 2006). A recent study has validated that variation in PRPF6 may result in assembly and the corresponding function dysregulation of colon cancer cell spliceosome, which may lead to cancer (Adler et al., 2014). Compared with NSCLC, SCLC grows faster and transfers earlier. Many studies also revealed that overexpressed SRSF3 can increase the expression of FOXM1, Cdc25B and PKL1 and promote cell growth through G2/M phrase (He et al., 2010; Jia et al., 2010; Kurokawa et al., 2014). Apart from that, TRA2B could induced BCL2 overexpressed which inhibited cell apoptosis (Kuwano et al., 2015). Combining proteomic data with GEO database, 14 DEPs that were positively correlated with their genes were selected for further investigation. The Reactome enrichment analysis indicated that nine of them were highly enriched in membrane-bounded vesicle and extracellular exosome.

The majority of these proteins have been proved to be associated with cancers. Among these nine proteins, ANXA2 is a membrane-bound protein that is usually relevant to cell invasion and metastasis (Lokman et al., 2011). It was reported that the survival rate of patients with lung cancer decreased when ANXA2 was up-regulated, which might serve as a potentail biomarker for NSCLC (Agababaoglu et al., 2017; Wang et al., 2012). In addition, down-regulation of ANXA2 could attenuate tumor growth and metastasis in lung cancer, which could reduce the size of lung cancer tumor to 19% (Andey et al., 2014). Another membrane-bound protein ANXA1 was reported to be involved in the cancer development as well. High abundance of ANXA1 was identified in patients with lung cancer while knockdown of ANXA1 can inhibit the proliferation, migration and invasion of NSCLC, especially in A549 cell line (Fang et al., 2016; Liu et al., 2011; Qiu et al., 2008). In addition, a clear interaction between ANXA2 and ANXA1 has been observed, which suggested that these two proteins might work together to promote the rapid proliferation of cancer cells. According to a recent study, both ANXA1 and ANXA2 could be up-regulated with the stimulation of GAS1 and induce the stagnation of the cell (Perez-Sanchez et al., 2018).

Based on a report published in 2015, knockdown of FLNB in A549 cell line resulted in slow down of invasion ability compared with normal A549 cell line. FLNB enhanced invasion of lung cancer cells through phosphorylation of MRLC and FAK (Iguchi et al., 2015). Therefore, FLNB might serve as a treatment target of NSCLC. HNRNPA2B1 from the A/B subfamily of ubiquitously expressed heterogeneous nuclear ribonucleoproteins (hnRNPs), was identified as a biomarker for early diagnosis of lung cancer (Sueoka et al., 2001) since it was overexpressed in NSCLC (Sueoka et al., 1999). Furthermore, HNRNPA2B1 could enhance COX-2 and promote NSCLC growth. Recent evidence indicated that knockdown of HNRNPA2B1 can inhibit the migration and proliferation of NSCLC (Xuan et al., 2016).

In addition, ME2 also played a crucial role in lung cancer growth. ME2 encodes a mitochondrial NAD-dependent malic enzyme which was highly expressed in lung cancer (Sarfraz et al., 2018). In A549 cell line, ME2 depletion can suppress the cell proliferation and induce the cell death and differentiation via affecting expression of PTEN and PDK1 and inhibiting the AKT pathway (Ren et al., 2014). APRT, a human metabolic enzyme, can lead to a significantly decreasing of leukaemia cell proliferating by inhibiting the synthesis of polyamines when it was knocked down (Pey et al., 2017).

In addition, the other eight proteins have also been reported in other types of cancers and may be closely associated with lung cancer proliferation or invasion. For instance, it has been validated high expression of Anp32E are associated with shorter survival time and high risk of disease relapse in Triple- negative breast cancer (Xiong et al., 2018). AKR1B1 has been validated as biomarkers in breast cancer (De Groot et al., 2014).

## Conclusion

In summary, with a combined use of quantitative proteomics analysis and their corresponding transcriptome data, we identified 14 DEPs among NSCLC and SCLC cell lines. Bioinformatics analysis indicated that these proteins are mainly involved in the cell migration, proliferation and invasion, and many of them has been reported to be associated with cancers. Due to the limited number of cell lines used, the results presented in this manuscript might still require further validations. Even through, this research still revealed important proteogenomic differences between NSCLC and SLCL cells, which can be complementary to existing genomic and proteomic data related to lung cancers and will be crucial for a systems biology-level understanding of the molecular mechanism of lung cancers.

##  Supplemental Information

10.7717/peerj.8779/supp-1 Supplemental Information 1 Supplementary Tables (S1) All proteins identified from four different NSCLC or SCLC cell lines. (S2) Quantitative proteins among four different cell lines with at least five PSMs. (S3) Differentially expressed proteins in small-cell lung cancer (SCLC) cell lines comparing with non-small-cell lung cancer cell lines (NSCLC). (S4) Fourteen differetially expressed proteins that positively correlated with their mRNA.

## Abbreviations

NSCLC: Non-Small Cell Lung Cancer
SCLC: Small Cell Lung Cancer
DEG: Differently Expressed Gene
DEP: Differently Expressed Protein
GO: Gene Ontology
KEGG: Kyoto Encyclopedia of Genes and Genomes
GEO: Gene Expression Omnibus

## Additional Information and Declarations

### Data Availability

The following information was supplied regarding data availability: The proteomic data are available from the PRIDE Archive: PXD015270.
